# Clinical factors associated with statins prescription in acute ischemic stroke patients: findings from the Lombardia Stroke Registry

**DOI:** 10.1186/1471-2377-14-53

**Published:** 2014-03-21

**Authors:** Isabella Canavero, Anna Cavallini, Patrizia Perrone, Mauro Magoni, Lucia Sacchi, Silvana Quaglini, Giordano Lanzola, Giuseppe Micieli

**Affiliations:** 1 Department of Emergency Neurology/Stroke Unit, National Neurologic Institute C. Mondino IRCCS, Pavia, Italy; 2 Neurology Unit, Legnano Hospital, Legnano, Italy; 3 Neurovascular Unit, ‘Spedali Civili’ Hospital, Brescia, Italy; 4 Department of Electrical, Computer and Biomedical Engineering, University of Pavia, Pavia, Italy

**Keywords:** Statins, Ischemic stroke, Adherence, Performance predictors, Classification tree

## Abstract

**Background:**

Statins, due to their well-established pleiotropic effects, have noteworthy benefits in stroke prevention. Despite this, a significant proportion of high-risk patients still do not receive the recommended therapeutic regimens, and many others discontinue treatment after being started on them. The causes of non-adherence to current guidelines are multifactorial, and depend on both physicians and patients. The aim of this study is to identify the factors influencing statin prescription at Stroke Unit (SU) discharge.

**Methods:**

This study included 12,750 patients enrolled on the web-based Lombardia Stroke Registry (LRS) from July 2009 to April 2012 and discharged alive, with a diagnosis of ischemic stroke or transient ischemic attack (TIA) and without contra-indication to statin therapy. By logistic regression analysis and classification trees, we evaluated the impact of demographic data, risk factors, tPA treatment, in-hospital procedures and complications on statin prescription rate at discharge.

**Results:**

We observed a slight increase in statins prescription during the study period (from 39.1 to 43.9%). Lower age, lower stroke severity and prestroke disability, the presence of atherothrombotic/lacunar risk factors, a diagnosis of non-cardioembolic stroke, tPA treatment, the absence of in-hospital complications, with the sole exception of hypertensive fits and hyperglycemia, were the patient-related predictors of adherence to guidelines by physicians. Overall, dyslipidemia appears as the leading factor, while TOAST classification does not reach statistical significance.

**Conclusions:**

In our region, Lombardia, adherence to guidelines in statin prescription at Stroke Unit discharge is very different from international goals. The presence of dyslipidemia remains the main factor influencing statin prescription, while the presence of well-defined atherosclerotic etiopathogenesis of stroke does not enhance statin prescription. Some uncertainties about the risk/benefit of statin therapy in stroke etiology subtypes (cardioembolism, other or undetermined causes) may partially justify the underuse of statin in ischemic stroke. The differences that exist between current international guidelines may prevent a more widespread use of statin and should be clarified in a consensus.

## Background

Statins have noteworthy benefits in stroke prevention as well as in cardiovascular diseases due to their well-established pleiotropic action [1]. In fact, beyond their well-known lipid-lowering effect, their role in the stabilization of endothelial function, in immunomodulation and antithrombotic processes, has been hypothesized and subsequently demonstrated [2-4]. After the SPARCL trial [5] many other studies have indicated that statins are of benefit to the majority of ischemic stroke patients as they reduce the risk of recurrence and improve outcome [6-13]. It has also been observed that daily intake of a statin after an acute ischemic stroke could improve functional outcome in patients with LDL levels ≤ 100 mg/dL [2]. The last Italian guidelines for stroke [14], in agreement with recommendations from other international guidelines [15,16], state that statins are indicated in patients with ischemic stroke even in the absence of hypercholesterolemia. Moreover, the most recent ACC-AHA guidelines for management of high blood cholesterol levels [17], based on RCTs results, recommend a high-intensity statin therapy for *every* patient with “clinical atherosclerotic CVD” (ASCVD). ASCVD patients include acute coronary syndromes, a history of myocardial infarction, stable or unstable angina, coronary or other arterial revascularization, stroke, TIA or peripheral atherosclerotic arterial disease. In the presence of at least one of these clinical events, patients should receive statin therapy regardless LDL cholesterol levels [18].

Despite this scientific evidence and guideline recommendations, an unacceptably high proportion of stroke patients are neither on lipid-lowering therapy nor managed aggressively enough to achieve recommended target cholesterol levels [19,20]. Causes for non-adherence to current guidelines are multifactorial, and depend both on physicians and patients.

Understanding the gap between a physician’s knowledge and his actual actions may be essential for the development of strategies aiming to improve patient management: predictors of adherence and causes for non-adherence should be identified and evaluated carefully.

Most studies have examined indirectly physicians’ adherence to guidelines, through self-administered questionnaires and interviews. To our knowledge, there have been only a few attempts to describe the topic using data from real clinical practice (i.e., the prescribed therapies as they appear on the discharge letter), and to characterize the clinical factors interfering with statin prescription: the GWTG Stroke, the Swedish Stroke Register, the Paul Coverdell National Stroke Registry [21-23]. These registries describe prescription trends but are not able to identify the barriers to prescription.

The aim of this study is to identify the clinical factors influencing statin prescription by physicians in acute ischemic stroke patients at discharge from Lombardia Stroke Units [24]. We analyzed data collected from a web-based registry: the Lombardia Stroke Registry (LSR), describing the activity of our region’s Stroke Units.

## Methods

The study was based on data collected from July 2009 to April 2012 at 42 Stroke Units participating in the LSR. The LSR gathers demographic, clinical, and procedural data of acute stroke patients. Data-entry was performed by external staff, after training on how to retrieve data from hospital-specific clinical charts and documentation. In 40 of the 42 Stroke Units the treating specialty is neurology, in one internal medicine and in one the management switched from internal medicine to neurology during the study period. All the physicians operating in our Stroke Units are certified for the administration of the NIHSS and the modified Rankin scale (mRS).

For this study, we considered only the patients discharged alive, with a diagnosis of ischemic stroke or TIA, and without clinical contra-indications to statin prescription (e.g. hepatopathy, myopathy or hemorrhagic diathesis). They were divided into 2 groups: ‘Statin +’ group = patients discharged on statin therapy and ‘Statin -’ group = patients not discharged on statin therapy.

### Clinical variables

The following variables were considered in the analysis: 1. demographics (age, gender); 2. prestroke and discharge disability (evaluated by mRS); 3. vascular risk factors and comorbidities (previous TIA/stroke, arterial hypertension, diabetes mellitus, dyslipidemia, atrial fibrillation, myocardial infarction, coronary artery disease, peripheral artery disease, smoking, heart failure, cognitive impairment, prosthetic cardiac valve); 4. stroke severity at admission and at discharge (evaluated by the National Institute of Health Stroke Scale - NIHSS score); 5. emergency treatment (intravenous or intra-arterial thrombolysis); 6. in-hospital neurological and medical complications (defined as the occurrence or absence of the following events: intracranial hypertension, hypertensive fits, seizures, bleeding, hypoxemia, hyperglycemia, falls, psychiatric disorders, deep venous thrombosis/pulmonary embolism, atrial flutter/fibrillation, acute myocardial infarction, bedsores, ventricular arrhythmias, fever, pneumonia, urinary infections); 7. stroke subtype (TIA or ischemic stroke) and etiopathogenetic classification according to TOAST criteria [25]; 8. statin and other secondary prevention therapies at discharge.

Risk factors/comorbidities were considered as present if the factor was documented in past medical history or the patient was on drug treatment for a specific comorbidity.

### Statistical analysis

Patients were coded as 1 = Statin + and 0 = Statin -. All the variables were coded as: 1 = present, 0 = absent. Age was codified as 0 if age < 80yrs and as 1 if age > = 80 yrs; gender was codified as 0 = female and 1 = male. mRS score was discretized into two levels: no disability (mRS < 2) and disability (mRS > =2). No disability was assigned the code 0 while disability was coded as 1. NIHSS score was recoded as 0 for values ≤ 8 and 1 for values >8. Differences between the two groups of patients, stratified for Statin + and Statin-, were analyzed by a Chi square test for categorical variables. To build a predictive model for statin prescription at discharge, a multivariate logistic regression was performed through a stepwise analysis. First, we performed two separate analyses for the prestroke and in-hospital variables, then we evaluated an overall model. All the analyses were performed with S-Plus [26]. The logistic regression model highlights the variables that are significant independent predictors of statin prescription. However, the variable combination leading to statin prescription is not explicitly identified, even if it could be useful in analyzing physicians’ motivations for non-adherence to guidelines. Thus, in addition to logistic regression we exploited machine learning (ML) techniques to describe the possible care paths that lead to a specific outcome (prescription/non-prescription). In particular, we applied the ML ‘Classification Tree’ algorithm to the set of variables identified by the logistic regression analysis.

Classification Trees are algorithms that work by recursively partitioning the examples space, based on the most discriminant feature in order to predict the ‘class variable’ (i.e. statin prescription). Thanks to their structure, Classification Trees allow easy visualization, exploration and interpretation of results. In our study, each branch of the tree identifies a set of conditions (e.g. age, risk factors, treatments) that define subsets of patients where statin prescription is more or less likely.

For the construction of Classification Trees we used the algorithm that is implemented in the Orange Data Mining Suite [27,28]. This study was approved by the Ethics Committees of all of the institutes co-operating in the Lombardia Stroke Network (see Acknowledgements for details). Written informed consent for the handling of personal data was obtained from all subjects.

## Results

Out of a total of 16,002 patients in the registry, 12,873 were discharged alive with a diagnosis of ischemic stroke or TIA. Out of these, 123 (0.95%) had contra-indications to the prescription of statins and were not included in the analysis. Thus, 12,750 patients meeting the inclusion criteria were analyzed: 5392 (42%) belong to the Statin + group and 7358 (58%) to the Statin – group. The percentage of patients discharged with statin therapy was 39.1% in 2009, 41.9% in 2010, 42.7% in 2011 and 43.9 % in 2012, showing a slight upward trend.

### Prestroke profiles

Table 1 was designed to study the predictive value of the individual patients’ demographic and prestroke clinical features. The table shows the percentage of statin prescription given the values of the different predictors considered. For example, out of 6779 males, 3107 (46%) underwent statin prescription. Out of (5971 = 12750–6779) females, 2285 (38%) underwent statin prescription. The percentage of prescription in males is significantly higher than that in females, as shown by the p-value in the last column.

**Table 1:** Statin prescription according to demographics, prestroke disability and risk factors/comorbidities

	**All (12750)**	**Statin + (5392, 42%)**		**p-value (χ**^**2**^**)**
**Gender,** n (%)	Male: 6779 (53%)	***Male***	***Female***	p < 0.0001
		3107 **(46%)**	2285 (38%)	
**Age,** n (%)	Age <80: 8889 (70%)	***Age < 80***	***Age ≥80***	p < 0.0001
		4251 **(48%)**	1141 (30%)	
**Prestroke mRS,** n^§^ (%)	mRS 0–1: 5719 (80%)	***mRS 0-1***	***mRS 2-5***	p < 0.0001
	mRS 2–5: 1469 (20%)	2649 **(46%)**	468 (32%)	
**Risk factors,** n (%)		***Rf present***	***Rf absent***	
Previous TIA/stroke	2931 (23%)	1271 (43%)	4121 (42%)	p = 0.18
Arterial hypertension	8166 (64%)	3628 **(44%)**	1764 (38%)	p < 0.0001
Diabetes mellitus	2619 (21%)	1284 **(49%)**	4108 (41%)	p < 0.0001
Myocardial infarction	1407 (11%)	832 **(59%)**	4560 (40%)	p < 0.0001
Hypercholesterolemia	2366 (19%)	1686 **(71%)**	3706 (36%)	p < 0.0001
Coronary artery disease	999 (8%)	546 **(55%)**	4846 (41%)	p < 0.0001
Peripheral artheriopathy	894 (7%)	462 **(52%)**	4930 (42%)	p < 0.0001
Atrial fibrillation	2150 (17%)	720 (34%)	4672 (44%)	p < 0.0001
Smoking	1804 (14%)	906 **(51%)**	4486 (41%)	p < 0.0001
Heart failure	460 (4%)	190 (41%)	5202 (42%)	p = 0.66
Cognitive impairment	967 (8%)	284 (29%)	5108 (43%)	p < 0.0001
Valvular prosthesis	313 (2%)	137 (44%)	5255 (42%)	p = 0.59

^§^Not available for all patients.

In addition to male gender, patients with the following characteristics were more likely to be treated with statins: lower age, lower prestroke mRS score, personal history of hypertension, diabetes, myocardial infarction (MI), dyslipidemia, coronary artery disease (CAD), peripheral artery disease (PAD), smoking, and absence of personal history of atrial fibrillation (AF) or cognitive impairment. At multivariate analysis, considering these prestroke variables, the independent ones predicting statin prescription at discharge were (sorted by the variable influence on prescription):

– personal history of dyslipidemia (OR 3.71, 95% CI 3.25-4.26, p value < 0.00001)

– personal history of myocardial infarction (OR 2.01, 95% CI 1.69-2.39, p value < 0.00001)

– age (OR 0.54, 95% CI 0.48-0.61, p value < 0.00001)

– personal history of PAD (OR 1.74, 95% CI 1.42-2.13, p value < 0.00001)

– prestroke mRS (OR 0.68, 95% CI 0.59-0.78, p value <0.00001)

– personal history of atrial fibrillation (OR 0.70, 95% CI 0.61-0.81, p value < 0.0001)

– personal history of CAD (OR 1.24, 95% CI 1.01-1.53, p-value = 0.04)

– personal history of smoking (OR 1.19, 95% CI 1.03-1.38, p value = 0.01)

– personal history of diabetes (OR 1.15 CI 1.02 - 1.31, p value = 0.02)

– personal history of hypertension (OR 1.15, 95% CI 1.04-1.28, p value = 0.008)

### In-hospital phase

As shown in Table 2, at the emergency department 5.0% (n = 636) of patients received thrombolysis treatment (544 patients were treated with intravenous thrombolysis and 92 with intra-arterial thrombolysis). Statins were prescribed in 294 (54%) patients who had been treated with intravenous thrombolysis, and in 37 (40%) patients treated with intra-arterial thrombolysis. Among patients who were not treated with thrombolysis, 5061 (42%) received statin prescription at discharge. These percentages are significantly different (p < 0.0001).

**Table 2:** Hospitalization: stroke severity, neurological and medical complications, disability at discharge

**Emergency treatment:**	**All (n = 12750)**	**Statin +**			**p-value**
Intravenous thrombolysis	544 (4.3%)	294 (54%)			p < 0.0001
Intra-arterial thrombolysis	92 (0.7%)	37 (40%)			
No thrombolysis	12114 (95%)	5061 (42%)			
**Stroke severity (NIHSS):**		***NIHSS*** ≤ ***8***	***NIHSS >8***		
**At admission** available in 8470 pts	NIHSS ≤ 8: 6199 (73%)	2846 (46%)	849 (37%)		p < 0.0001
	NIHSS >8: 2271 (27%)				
**At discharge** available in 8132 pts	NIHSS ≤ 8: 6691 (82%)	3171 (47%)	544 (38%)		p < 0.0001
	NIHSS >8: 1441 (18%)				
**mRS at discharge** available in 7958 pts	mRS 0–1: 3487 (44%)	***mRS 0-1***	***mRS 2-5***		p < 0.0001
	mRS 2–5: 4471 (56%)	1707 (49%)	1847 (41%)		
**In-hospital complications, n (%)**		***Present***	***Absent***		
Intracranial hypertension	427 (3.5%)	170 (40%)	5222 (42%)		p = 0.29
Acute myocardial infarction	37 (0.3%)	22 (59%)	5370 (42%)		p = 0.03
Seizures	270 (2%)	91 (34%)	5301 (42%)		p = 0.004
Hypoxemia	782 (6%)	250 (32%)	5142 (43%)		p < 0.0001
Hypertensive fits	2240 (18%)	1060 (47%)	4332 (41%)		p < 0.0001
Hyperglycemia	1136 (9%)	579 (51%)	4813 (41%)		p < 0.0001
Atrial fibrillation/flutter	931 (7%)	324 (35%)	5068 (43%)		p < 0.0001
Fever	1922 (15%)	683 (35%)	4709 (43%)		p < 0.0001
Ventricular arrhythmia	57 (0.5%)	24 (42%)	5368 (42%)		p = 0.97
Bedsores	195 (1.5%)	40 (20.5%)	5352 (43%)		p < 0.0001
Deep venous thrombosis/pulmonary embolism	49 (0.4%)	16 (33%)	5376 (42%)		p = 0.17
Bleedings	547 (4%)	182 (33%)	5210 (43%)		p < 0.0001
Urinary infections	1276 (10%)	431 (34%)	4961 (43%)		p < 0.0001
Falls	158 (1%)	69 (44%)	5323 (42%)		p = 0.72
Pneumonia	419 (3%)	130 (31%)	5262 (43%)		p < 0.0001
Psychiatric disorders	488 (4%)	193 (39.5%)	5199 (42%)		p = 0.21
**Diagnosis at discharge**		***Ischemic stroke***	***TIA***		
Ischemic stroke/TIA	10534 (83%)	4482 (43%)	906 (41%)		p = 0.14
**TOAST classification** available in 8564 patients		***All***	With other indications to prescription*	***Without other indications to prescription***	
Cardioembolism (possible/probable)	3083 (36%) With* other indications to prescription: 1059; without 2024	1113 (36%)	542 (51%)	571 (28%)	p < 0.0001
Non-cardioembolism:	5481 (54%) With* other indications to prescription: 1819; without 3662	2607 (47%)	1185 (65%)	1422(39%)	
-Large vessels atherosclerosis	2078 (24%) With* other indications to prescription: 766; without 1312	1077 (52%)	504 (66%)	573 (44%)	
-Small vessels disease	1983 (23%) With* other indications to prescription: 668; without 1315	979 (49%)	458 (68%)	521 (40%)	
-Other causes	178 (2%) With* other indications to prescription: 43; without 135	48 (27%)	24 (56%)	24 (18%)	
-Undetermined etiology	1242 (15%) With* other indications to prescription: 342; without 900	503 (40%)	199 (58%)	304 (34%)	
**Antithrombotic treatment at discharge**					
Antiplatelet	9763 (76%)		4296 (44%)		p < 0.0001
Anticoagulant	2130 (17%)		933 (42.8%)		
None	857 (7%)		163 (19%)		

* = dyslipidemia, myocardial infarction, coronary artery disease (CAD), peripheral artery disease (PAD).

Patients with less severe neurological deficits, both at admission and at discharge, and minor disability at discharge, are more likely to receive statin prescription. The occurrence of complications during hospitalization (except for acute myocardial infarction, hypertensive fits and hyperglycemia) seems to lower the statin prescription rate. At discharge, the distribution of statin prescription was similar for ischemic stroke patients and TIAs. An etiopathogenetic diagnosis of cardioembolism made it less likely to have statin prescribed at discharge.

**Figure 1:**
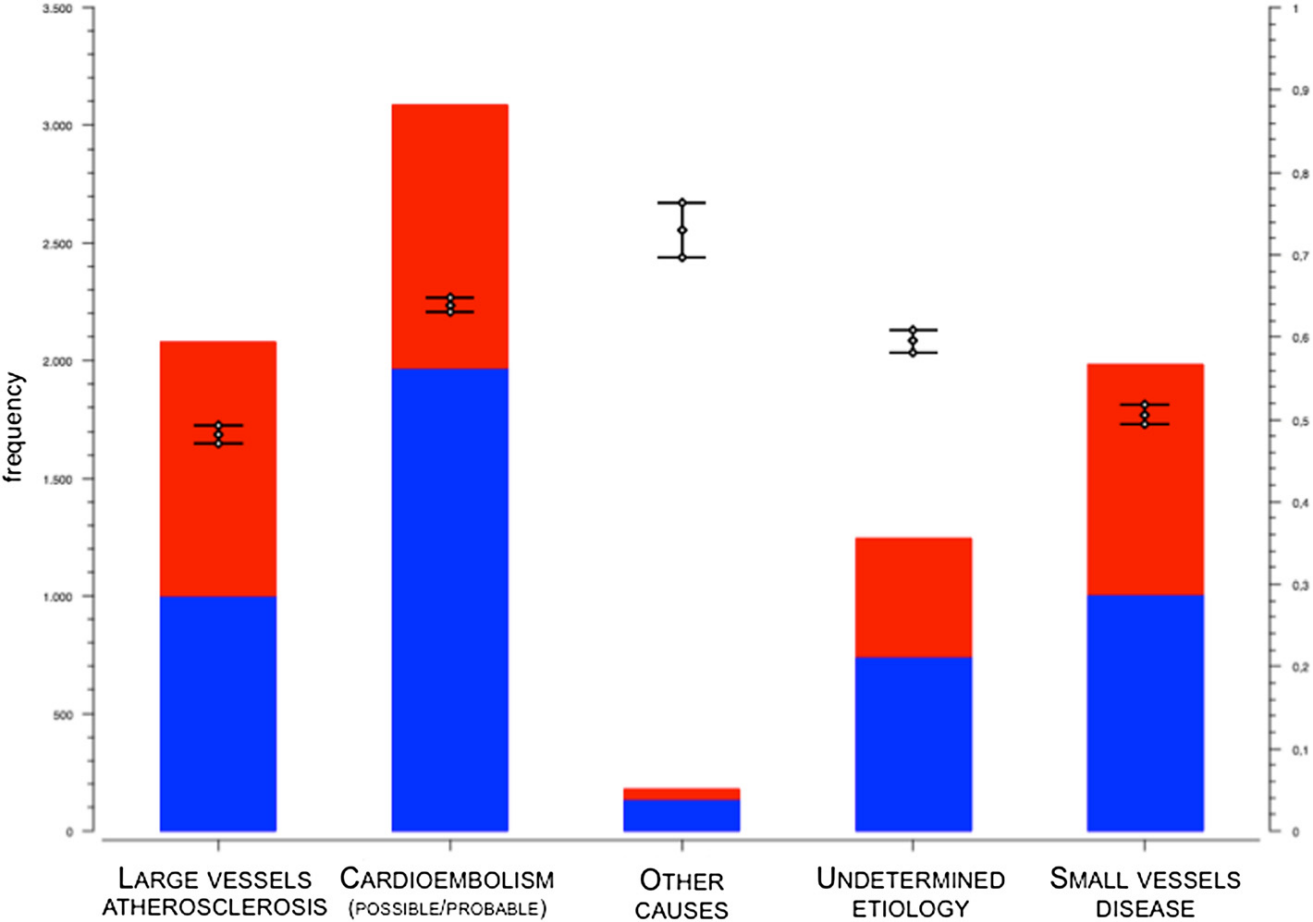
Statin prescription (red: presence, blue: absence) according to stroke etiology.

Considering the different TOAST etiopathogenesis, from Figure 1 we can see that statin prescription is low in all the diagnosis subtypes.

Comparing antithrombotic treatment at discharge, patients who did not receive any antithrombotics were less likely to be treated with statins.

At multivariate analysis, the in-hospital variables supporting statin prescription at discharge were (sorted by the variable influence on prescription):

– antithrombotic therapy at discharge (OR 2.65, 95% CI 2.02-3.48, p-value < 0.00001)

– thrombolysis (OR 2.18, 95% CI 1.69-2.81, p value < 0.00001)

– pneumonia (OR = 0.64, 95% CI 0.47-0.89, p-value = 0.008)

– hyperglycemia (OR 1.5, 95% CI 1.22-1.84, p value < 0.0001)

– intracranial hypertension (OR = 1.42, 95% CI 1.04- 1.96, p-value = 0.03)

– cardioembolism (OR = 0.72, 95% CI 0.64-0.82, p-value < 0.00001)

– urinary infections (OR = 0.75, 95% CI 0.62-0.92, p-value = 0.006)

– hypertensive fits (OR 1.29, 95% CI 1.12-1.5, p value = 0.0006)

– NIHSS at admission (OR 0.80, 95% CI 0.67-0.96, p value = 0.016)

– mRS at discharge (OR = 0.87, 95% CI 0.77-0.99, p-value = 0.04)

### Predictors of statin prescription at discharge

Considering all the variables without distinguishing between prestroke and in-hospital phases, we obtained the following overall stepwise logistic regression model (sorted by the variable influence on prescription):

– personal history of dyslipidemia (OR 3.88, 95% CI 3.3 - 4.56, p value < 0.00001)

– antithrombotic therapy at discharge (OR 2.72, 95% CI 2.03-3.66, p-value < 0.00001)

– personal history of myocardial infarction (OR 1.92, 95% CI 1.56-2.37, p value < 0.00001)

– age (OR 0.55, 95% CI 0.48-0.64, p value < 0.00001)

– personal history of PAD (OR 1.64, 95% CI 1.29-2.09, p value < 0.0001)

– thrombolysis (OR 1.64, 95% CI 1.24-2.17, p value = 0.0005)

– pneumonia (OR = 0.64, 95% CI 0.44–0.91, p-value = 0.015)

– hypoxemia (OR 0.69, 95% CI 0.52-0.92, p value = 0.01)

– personal history of atrial fibrillation (OR 0.73, 95% CI 0.6-0.89, p value = 0.002)

– prestroke mRS (OR 0.78, 95% CI 0.67-0.92, p value = 0.003)

– hypertensive fits (OR 1.28, 95% CI 1.09-1.51, p value = 0.002)

– NIHSS at discharge (OR 0.79, 95% CI 0.66-0.95, p value = 0.013)

– hyperglycemia (OR 1.22, 95% CI 0.96-1.55, p value = 0.01)

– personal history of diabetes (OR 1.2 CI 1.03-1.41, p value = 0.02)

– cardioembolism (OR = 0.86, 95% CI 0.73-1, p-value < 0.06)

**Figure 2:**
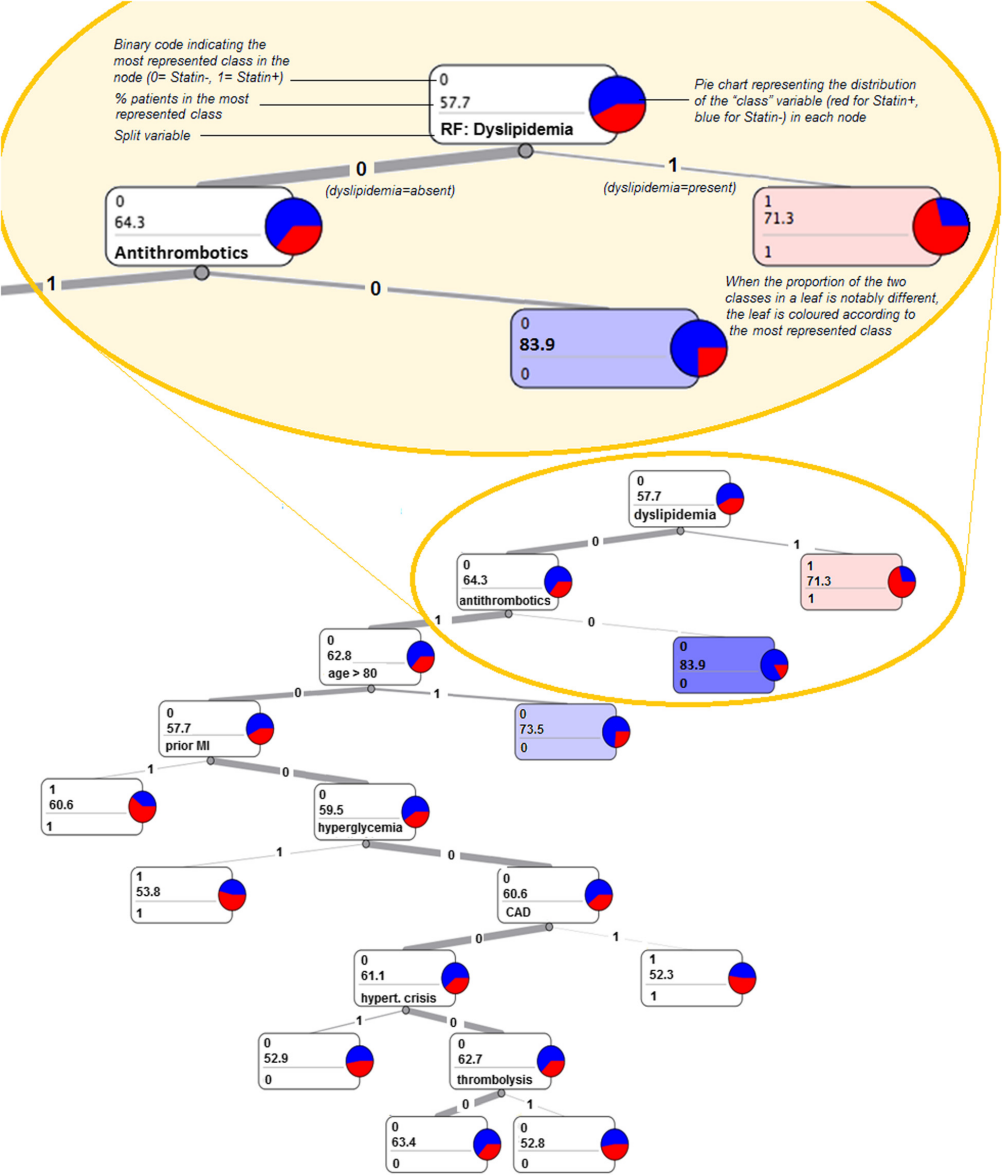
**The classification tree graph.** (legend) The classification tree obtained using the most predictive variables according to the logistic regression analysis, and depicting paths leading to higher/lower prescription probability. The yellow box explains how to read a classification tree graph. The root node shows that, in the whole sample, the most represented class is Statin- (57.7%). Moreover, it shows that the most important variable distinguishing the two classes is Dyslipidemia: when present, 71.3% of patients receive statins at discharge; when absent, only 35.7% (100–64.3) of patients undergo the prescription.

Figure 2 shows the Classification Tree obtained using the variables selected by the overall logistic regression analysis. This picture can be used in order to better explain the analysis results.

Dyslipidemia, at the top of the graph, is the most informative parameter in predicting statin prescription. If present, statins are prescribed in more than 70% of cases.

In the absence of dyslipidemia, the most informative indicator is the prescription of antithrombotic therapy: about 84% of the patients who are not undergoing antithrombotic treatment are not undergoing statin prescription either.

Also for patients under antithrombotic treatment, Statin- is the majority class. However, the tree shows three groups of patients where Statin + is the majority class: patients aged less than 80 who had a prior MI, those who experienced hyperglycemic episodes during the hospital stay and those affected by CAD.

### Subgroup analysis

**Figure 3:**
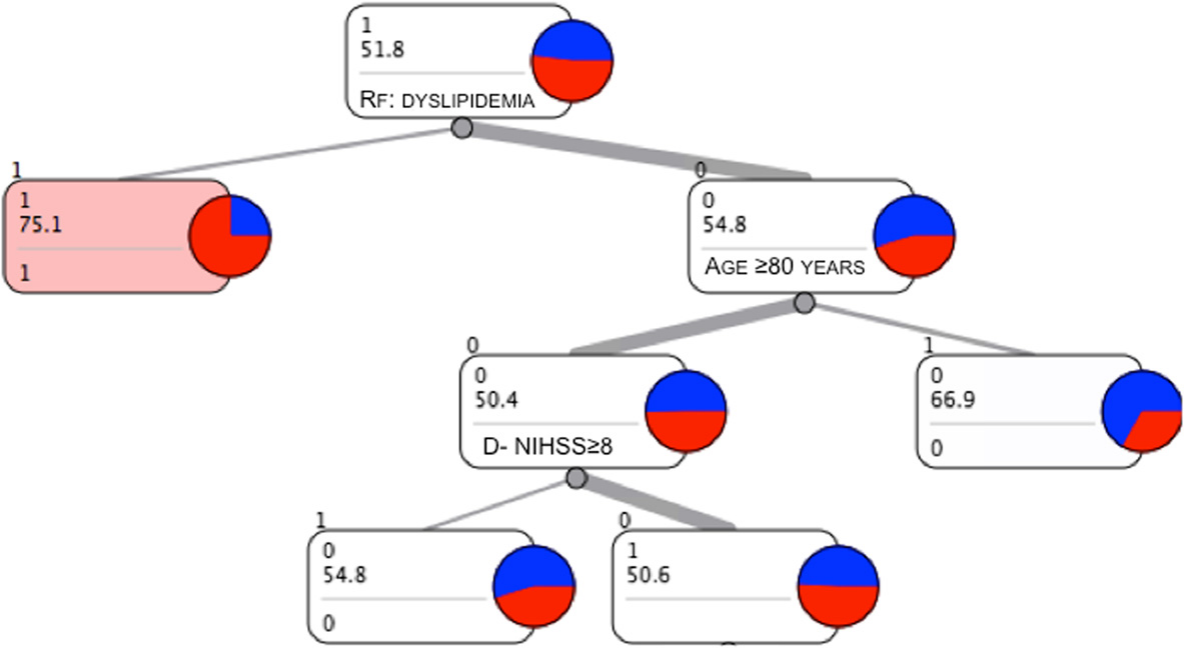
Classification tree graph for the subgroup Large Vessels Disease (LVD).

Considering the almost equal distribution of statin prescription across TOAST groups (Figure 1), we were not expecting TOAST classification to appear in the top portion of the classification tree. However, since the presence of Large Vessels Disease (LVD) is the main indication for statins and several works including the SPARCL trial [5] consider patients with stroke/TIA with non-cardioembolic stroke, we investigated this group of patients in a separate classification tree. As shown in Figure 3, the leading factor in statin prescription is the presence of dyslipidemia, as it is for the whole population.

Considering LVD patients without dyslipidemia (right branch of the tree), the algorithm identified age as the most informative parameter in the following decision process. In the majority of elderly patients, statins are not prescribed while for younger patients, the proportion of statin prescription and statin non-prescription is almost identical. Thus, from our data, we cannot identify any factors determining the choice of whether or not to prescribe statins in younger patients without dyslipidemia, even in the presence of well-defined atherosclerosis.

## Discussion

In the literature there is a growing body of papers concerning the factors that can influence physicians’ attitude to the prescription of new drugs. Statins are, in fact, quite old drugs, whose range of clinical indications has broadened after the recent RCTs.

After the publication of the SPARCL trial results in 2006, a progressive increase in the number of patients who receive statin therapy at discharge in the United States has been reported. In the GWTG-Stroke study, the percentage of statin prescription increased from 75.7% in 2005 to 83.5% in 2007, however it was not considered high enough [21]. In our study, the percentage of patients treated with statins is only 42%, with a slight increase during the study period. Few other settings show worse results than ours. For example, in a recent meta-analysis conducted in China, only 24% of ischemic stroke patients were treated with statins [29]. It has been hypothesized that epidemiological features of Chinese people – lower mean blood cholesterol levels, more frequent hemorrhagic complications than Western people – could play a role in physicians’ decision-making process.

Prescription decisions are affected by many factors related to physicians’ attitudes and patient characteristics. Concerning physician-related factors, some authors have identified as good predictors of adherence to guidelines the habitual reading of medical journals and maintenance of professional contacts, such as talking to colleagues and attending scientific meetings [30]. It is also widely recognized that new drugs and guidelines are adopted earlier by specialists [30] and that clinical trial investigators are usually more prone to follow guidelines because of their link to the world of research [31]. Another study describes the so-called ‘therapeutic traditions’ which seem to influence the prescription behavior of individual physicians [32]. All these factors are difficult to quantify. As for patient-related factors, those able to influence their attitude to comply with therapy (after discharge) are usually identified in the literature as patients’ demographic, social and cognitive features. On the contrary, clinical factors detected during hospitalization are not usually considered as being among those that influence physicians’ prescription.

To overcome this limitation, our work tried to identify those patient-specific clinical and care factors that are able to influence physicians’ decisions to prescribe statin after an acute ischemic stroke. In our study, patients were more likely to be treated with statins if they were younger than 80 years old and not disabled before stroke, they had a personal history of risk/disease of systemic atherosclerosis (dyslipidemia, diabetes, myocardial infarction, coronaropathy, peripheral arteropathy), they were treated with thrombolysis at the emergency department and discharged on antithrombotics for the secondary prevention of ischemic stroke.

It is interesting to note that, in our study, dyslipidemia represents the principal factor leading to the prescription of statin in ischemic stroke of all subtypes, including in LVD stroke.

Among demographics, older age, previous disability and lower social status, have been previously reported to negatively influence the adherence to guidelines in many settings [33]. According to the Swedish Stroke Register [22] the prescription of statins was strongly age-related: statin therapy, even if associated with reduced risk of death, seemed to be underused among elderly patients, with an inverse correlation with increasing age. In line with these findings, in our setting younger patients and those without prestroke disability were more likely to be treated with statins. A higher risk of hemorrhagic complications in older people could explain this trend, even if other authors have not found a different rate of side effects compared to younger patients [34]. It has been suggested that older people [35] and people with prior disability [36] tend to discontinue treatment during follow up. The hypothetical reduced compliance could, at least partially, explain the low frequency of prescription in these subjects. Another important point is that most of the current evidence derives from RCTs based on patients with an average age of 65 years or younger and little comorbidity [5]. Thus, the observed lower prescription rate in older and disabled patients may perhaps be a consequence of this signaling. Moreover, current literature on statins efficacy shows controversial results: a recent work examined the impact of statins on outcome in older ischemic stroke patients, finding benefits in functional outcome and 12-month survival [34]; another paper analyzed the effect of statins in the elderly in the prevention of CV events, finding a lower rate of MI and stroke but worse survival rate [37]. Regarding gender, it has been already reported that female patients are often undertreated [33,38]; our study confirms this trend. Probably, the well-known higher risk of cardio-cerebrovascular pathologies in men than in women plays a role in the prescription-making process, even if it cannot completely justify it.

Stroke severity, directly linked to disability and life expectancy, is another factor our model found as influencing prescription. Patients with lower NIHSS scores both on admission and at discharge are more likely to receive statins. These findings suggest that physicians tend to prescribe statins in patients with milder neurological damage, perhaps in order to obtain better compliance. Higher severity could thus represent a 'non-prescription' bias leading physicians to avoid secondary preventive therapies in patients who have had severe strokes and with poor prognosis. Then, as already pointed out, patients with significant disability and poor prognosis are excluded from the dedicated RCTs, whose results cannot be applied to them. Another possible explanation could be linked to the highest risk of hemorrhagic transformation characterizing patients with more severe neurological deficits [39]. A post hoc analysis of the SPARCL trial results found that the use of statins in stroke prevention was associated with an increase in the number of patients having hemorrhagic stroke [40]. Therefore, another factor in patients with higher stroke severity being less frequently treated with statins is the possibility of increased risk of hemorrhagic transformation in patients who are already susceptible.

We also found that patients who did not receive antithrombotics at discharge showed a low statin prescription rate. This could happen in a context of global non-adherence to guidelines. Alternatively, they could be untreated with antithrombotics because of a higher clinical severity (large infarction size, hemorrhagic transformation, etc.): probably physicians tend to avoid statin prescription in order to limit the possible higher risk of bleeding in the subgroup.

The presence of non-cardioembolic risk factors was among the main drivers of increased statin use in the GWTG-Stroke trial [21]: our results were in line with it. It is interesting to note that patients with hypertension and diabetes are more likely to be treated with statins than patients who are not affected by these risk factors, even if more than a half of them do not usually receive the prescription (see Table 1).

In the GWTG setting, patients with known dyslipidemia or who were on a cholesterol reducer at the time of hospital admission seem to be more likely to leave the hospital on a statin [21]. In our study too, comparing ORs, dyslipidemia shows the strongest correlation with statin prescription, followed by myocardial infarction and PAD; the Classification Tree confirmed dyslipidemia as the leading factor in prescriptive decisions. This means that dyslipidemia and cardiovascular pathologies still appear crucial in determining the need for statin therapy. Does this mean that stroke is not *per se* recognized as a vascular disease?

However, the relationship between total cholesterol levels and stroke outcome has not, as yet, been well characterized. Despite much clinical data supporting a linear relation between cholesterol lowering and cardiovascular risk reduction [41], in some papers hypercholesterolemia has been associated with better stroke outcome [42]. Recent works have described a ‘U-shaped’ association between cholesterol levels and stroke mortality, which was higher for patients with < 160 mg/dl [43]; the same cut-off was identified as a risk factor for a hemorrhagic fatal stroke [44].

Current guidelines for stroke [14-16] recommend statins for all patients with non-cardioembolic stroke. The best evidence from the literature derives from the SPARCL trial subanalysis: statins are mostly effective in reducing the risk of vascular events in patients with carotid atherosclerosis [45]. The last ACC-AHA guidelines [17] for lipid management identify “patients with clinical atherosclerotic cardiovascular disease” (including TIA and stroke) as the first subgroup of patients that should receive a high-intensity statin therapy, if tolerated.

In our setting, statin prescription rates for cardioembolism and ‘other causes’ of stroke are in line with current guidelines. However, it is particularly surprising that patients with large vessel disease, in the absence of other clinical indications for statins, have a prescription rate of only 44%, despite the current guidelines strongly recommending statin therapy for atherosclerotic disease. The Classification Tree applied to the subgroup of LVD patients leads us to conclude that dyslipidemia is the leading factor for statin prescription, even in the presence of well-defined atherosclerosis, instead of recognizing LVD per se as the clinical indication for statin reception.

Concerning small vessel disease, the low rate of prescription we detected could be explained by the complex relationship between statins and lacunar stroke, and by the fact that literature lacks precise indications. Small vessel disease determines both ischemic (lacunar) and hemorrhagic (microbleeds) stroke, and severe arterial hypertension is the main risk factor in both cases. According to these factors, statins could somehow enhance the risk of hemorrhage in patients with lacunar stroke because of the underlying small vessel pathology and unbalanced hypertension. Recent papers have analyzed the connections between statins use and the risk of developing microbleeds, with non-univocal results. Day et al. [46] found that previous statin therapy was not associated with the prevalence or degree of microhemorrhages in patients with acute ischemic stroke or transient ischemic attack. Haussen et al. [47] found instead that statins use was independently associated with microbleeds in patients with ICH. Some authors [48] underline that patients with non-atherosclerotic causes of stroke should not be treated with statins and that statin therapy should be initiated only after a careful consideration of all the potential risks and benefits that could derive from them.

Besides the low rate of prescription in atherosclerotic disease, the Classification Tree does not highlight the TOAST criteria among the predictors of statin prescription: the therapy is prescribed mostly according to the risk profile.

Unexpectedly, thrombolytic treatment in the acute phase of stroke seems to enhance statin prescription. Results of studies about the association between statin therapy and thrombolysis are somehow controversial. Some studies analyze the effect of statin treatment before stroke and functional outcome after thrombolysis, with non-coherent findings: some have found a positive influence from statins [49], while others do not [50-52]. Engelter et al. [52] found that prior statins use was not an independent predictor of functional outcome or of ICH after thrombolysis, but it may be considered as an indicator of baseline characteristics that are associated with a less favorable course. Cappellari et al. [53,54] suggested that statin treatment initiated within 24 hours of IV thrombolysis, but not statin treatment started before stroke and continued in the acute phase, may positively influence functional outcome. It is important to note that most of papers analyze the relationship between prior statin therapy and the clinical outcome after thrombolysis while our work describes the rate of statin prescription after thrombolysis. Our results could be explained with Cappellari’s findings. Further, to our knowledge, there is no evidence proving an increased risk of hemorrhage in patients treated with both rtPA and statins. We could also hypothesize that patients treated with thrombolysis are more likely to be managed following guidelines, and this could perhaps explain why they are more likely to receive the recommended statin regimen at discharge.

In the literature, statin therapy is mostly analyzed when administered during in-hospital stays. In this setting, it seems to be associated with a lower rate of complications in stroke patients. The prophylactic administration of statins significantly reduces in-hospital mortality [49] and is associated with a favorable outcome [55,56], with few incidences of adverse reaction [57]. Montaner et al. [58] reported that statin use between 3 and 12 hours after stroke was associated with neurological improvement at 3 days from ischemia, suggesting a rapid mechanism of action on coagulation or the fibrinolysis system. These findings should enhance physicians’ attitude to statin prescription. Considering our available data (i.e., statin prescription rate at discharge), however, we cannot hypothesize that statins could determine a lower occurrence of complications, but that patients with in-hospital complications are unlikely to be treated with statins, the only exception being hypertensive fits and hyperglycemia. This finding could be explained as previously done for age, disability and stroke severity: physicians avoid prescribing statins in critical patients, aside from situations that require statins for other indications than stroke. Besides, even if not confirmed by the logistic regression model, in our setting statins are not usually prescribed in patients with in-hospital bleeding, probably due to the higher risk of hemorrhage associated with statin use in the literature [59].

Besides these reports from the literature, we have to note that the effects of statins in acute stroke have been extrapolated - often with uncertain results - especially from retrospective studies, while few randomized clinical trials are currently available [60]. Consequently, current guidelines lack of some precise information, in particular concerning: their indication in non-atherothrombotic stroke [48], their recommended dose [60], the time to start therapy [8,58-62], their rate of side effects [12,40,57], the absolute contra-indications [48], the other concomitant therapies [12,49-53], the recommended approach with respect to old age (in RCTs concerning secondary prevention with statins the mean age is <65 years [12]).

In this paper we have not explored the physician-related factors influencing statin prescription but, reviewing the literature in order to compare our results with other experiences, we found more than a few *grey areas* among recommendations. We can hypothesize that the low statin prescription rate by physicians may be partially due to these controversies.

### Limits of the study

This study has some limitations. First, patient factors such as patient preferences and agreement to treatment are not addressed. As a matter of fact, these variables are not usually collected in a registry, and thus this information was not available in this study. Also, this study only provides the categorical data of vascular risk factors (e.g. diabetes mellitus, dyslipidemia, etc.) rather than the raw values of the biological parameters (e.g. blood glucose, total cholesterol, HDL, LDL etc.). The LSR Registry is a repository where mostly “qualitative” and process information is stored, focusing on the presence of a diagnosis rather than on its entity. Then, type and dosage of statin is not precisely reported in LSR, which may cause potential bias in statistical analysis. This is due to the Registry structure, modeled on the form of current clinical practice guidelines for stroke that in fact are not specific as well, recommending to use a statin without specifying which type.

Moreover, we performed the analyses without considering the type of providing treatment specialist, which might impact on the prescription decision-making. The reason why we did not consider such parameter is that the distribution of treatment specialty is very uneven, with only one center managed by internal medicine specialists, one whose management shifted during the study period and 40 managed by neurologists. In the internal medicine Stroke Unit, the percentage of statin prescriptions was higher (65.7%) than the overall average percentage (42%), in the one where the management shifted from Internal Medicine to Neurology it was slightly above the average (43.14%). As regards the centers managed by neurologists, the statins prescription rate has a high variability, with values ranging from 15.8% to 82.8%. From this data it is difficult to evaluate the role of the treating specialty as a predictor of prescription.

Although our Registry is provided with a follow-up section, in this study we have not included information on post-discharge statins use. The motivation underlying this choice is twofold. On the one hand, the follow-up is only partially completed for our patients. On the other hand, for those patients who died during follow-up, the registry doesn’t allow physicians to enter information about treatments in use before death. This would cause a bias, as the patients who died would result as not continuing the treatment even if they were.

## Conclusions

Our setting confirmed that adherence to guidelines regarding statin prescription rates by physicians is very different from international goals. Analyzing the decision-making process, we found that the presence of dyslipidemia - or at least a cardiovascular comorbidity - appears as the main factor influencing statin prescription, even if current guidelines recommend the drug in any case of non-cardioembolic stroke. On the contrary, the presence of well-defined atherosclerosis does not enhance statin prescription. Some uncertainties about the risk/benefit of statin therapy and *grey areas* in certain subgroups of stroke patients may partially justify the low rate of prescription we detected.

These findings must be considered in devising approaches to enhance adherence to guidelines by physicians to improve the management of patients and compliance to the recommended therapies. We highlight the need for a consensus on the above-mentioned *grey areas* in order to achieve improved and detailed guidelines that are easier for physicians to put into practice.

## Competing interests

No financial or non-financial competing interests to declare.

## Authors’ contributions

IC participated in the design of the study, in the statistical analysis, in review of the literature and drafted the manuscript. AC participated in the design of the original database, in the design of the study, in the statistical analysis, in the review of the literature and revised the manuscript. PP participated in the acquisition and interpretation of data. MM participated in the acquisition and interpretation of data. LS participated in the acquisition and interpretation of data and performed the statistical analysis (especially for Machine Learning techniques) and contributed to the manuscript drafting and revision. SQ participated in the design and realization of the original database, in the acquisition and interpretation of data and performed the statistical analysis and contributed to the manuscript drafting and revision. GL participated in the design and realization of the original database, in the acquisition of data and in coordinating the LSR centers. GM participated in the design of the original database, conceived the study, participated in coordinating the LSR centers, and revised the manuscript. All authors read and approved the final manuscript.

## Pre-publication history

The pre-publication history for this paper can be accessed here:

http://www.biomedcentral.com/1471-2377/14/53/prepub
